# Cycling using functional electrical stimulation therapy to improve motor function and activity in post-stroke individuals in early subacute phase: a systematic review with meta-analysis

**DOI:** 10.1186/s12938-023-01195-8

**Published:** 2024-01-02

**Authors:** Wagner Rodrigues Galvão, Luana Karoline Castro Silva, Magno Ferreira Formiga, George André Pereira Thé, Christina Danielli Coelho de Morais Faria, Ramon Távora Viana, Lidiane Andréa Oliveira Lima

**Affiliations:** 1 Master Program in Physiotherapy and Functioning, Federal University of Ceará, Fortaleza, Brazil; 2 Department of Teleinformatics Engineering, Federal University of Ceará, Fortaleza, Brazil; 3 Department of Physiotherapy, Federal University of Minas Gerais, Belo Horizonte, Brazil; 4 Department of Physiotherapy, Federal University of Ceará, Fortaleza, Brazil

**Keywords:** Stroke, Electrical stimulation, FEST, Gait, Systematic review

## Abstract

**Background:**

Stroke necessitates interventions to rehabilitate individuals with disabilities, and the application of functional electrical stimulation therapy (FEST) has demonstrated potential in this regard. This study aimed to analyze the efficacy and effectiveness of cycling using FEST to improve motor function and lower limb activity in post-stroke individuals.

**Methods:**

We performed a systematic review according to the recommendations of the PRISMA checklist, searching MEDLINE, Cochrane, EMBASE, LILACS, and PEDro databases by July 2022, without any date or language limitations. Studies were selected using the following terms: stroke, electrical stimulation therapy, cycling, and clinical trials. Randomized or quasi-randomized clinical trials that investigated the effectiveness of cycling using FEST combined with exercise programs and cycling using FEST alone for motor function and activity in subacute post-stroke individuals were included. The quality of included trials was assessed using the PEDro scores. Outcome data were extracted from eligible studies and combined in random-effects meta-analyses. The quality of evidence was determined according to the Grading of Recommendations Assessment, Development, and Evaluation system.

**Results:**

Five randomized clinical trials involving 187 participants were included. Moderate-quality evidence indicates that cycling using FEST combined with exercise programs promotes relevant benefits in trunk control (MD 9 points, 95% CI 0.36–17.64) and walking distance (MD 94.84 m, 95% CI 39.63–150.05, *I* = 0%), the other outcomes had similar benefits. Cycling using FEST alone compared to exercise programs promotes similar benefits in strength, balance, walking speed, walking distance, and activities of daily living.

**Conclusion:**

This systematic review provides low- to moderate-quality evidence that cycling using FEST may be an effective strategy to consider in improving motor function and activity outcomes for post-stroke individuals in the early subacute phase.

*Review registration*: PROSPERO (CRD42022345282).

**Supplementary Information:**

The online version contains supplementary material available at 10.1186/s12938-023-01195-8.

## Introduction

Stroke is currently the leading cause of disability in the world [1]. It is estimated that about 50% of survivors live with motor disabilities [2], affecting their level of physical activity and the development of comorbidities [3]. Advances in the acute treatment of stroke have led to reduced mortality rates and increased disability rates [4]. Clinical guidelines highlight the importance of rehabilitation strategies aimed at motor recovery in these individuals, promoting cost reductions in health care and increased social participation [5]. Strength and trunk control are considered predictors of independent walking in the post-stroke period [6]. Furthermore, walking speed and walking distance are fundamental to classifying the level of functional limitation, prediction of social participation, and functional Independence [7]. These outcomes are susceptible to change, especially in the initial phases after the stroke [8].

According to Langhorne et al. [9], the phases of stroke recovery are categorized into phase: hyperacute (0–24 h), acute (1–7 days), early subacute (7 days to 3 months), late subacute (3–6 months), and chronic (≥ 6 months). Adopting rehabilitation strategies in the first three months after a stroke promotes a greater chance of motor recovery beyond the expected spontaneous recovery and is considered a favorable period for rehabilitation [10]. In this period, there is spontaneous recovery associated with greater compensatory adaptation to physical training [11]. Spontaneous recovery comes from the remodeling of cortical structures [12] and neural receptors [13], in addition to changes in gene expression, among them the brain-derived neurotrophic factor, which is associated with neuroplasticity and motor learning [14]. Physical training promotes compensatory adaptations through the acquisition, retention, and consolidation of motor skills [11]. Thus, in addition to expecting improvement due to spontaneous recovery by restoring endogenous mechanisms, we must focus efforts on effective rehabilitation strategies in the early post-stroke periods.

Functional electrical stimulation has been recommended in the motor recovery of post-stroke individuals [5]. Recently, Marquez-Chin and Popovic [15] defined Functional Electrical Stimulation Therapy (FEST) as a promising intervention to aid or restore the ability of voluntary movements of individuals with motor impairments. FEST combines electrostimulation with specific task training, such as walking, reaching, grasping, and cycling [15, 16]. To be considered as FEST, this intervention must include three fundamental components: first, a patient must be actively attempting a functional motor task; second, the functional electrical stimulation current facilitates movement and generates sensory feedback; third, a therapist guides the limb in motion to ensure the quality and correctness of the movement. The therapist also adjusts the stimulation according to the changes observed in the patient throughout rehabilitation [15]. In the presence of active movements, there is sufficient activation of muscle spindles, Golgi tendon organs, and sensory receptors in the individual. Conversely, passive movements lack sensory feedback, thereby impairing the motor learning process [16]. It is therefore recommended that post-stroke individuals cycle with the assistance of functional electrical stimulation, promoting repeated voluntary stimuli and muscle activation in sequence which, over time, facilitates sensorimotor reorganization and adaptation, in line with the principles of neuroplasticity and highlighting the possible effectiveness of FEST [15, 16].

In post-stroke individuals, cycling using FEST has shown promising results related to strength [17, 18], walking speed [18, 19], walking distance [17–19], and balance [18]. Recently, a systematic review [20] evaluated the cycling induced by functional electrical stimulation compared to usual care in subacute post-stroke individuals. The authors concluded that cycling induced by functional electrical stimulation is not superior to conventional treatment for the outcomes of lower limb muscle function, tone, maintenance of standing position, and basic activities of daily living. Short-distance walking and sitting balance showed a statistical difference, but this was not considered clinically relevant. Nonetheless, this study [20] included two studies [21, 22] whose cycling was carried out using a robotic system called passive cycling, in which the equipment determined the cycling cadence, devoid of any active input or effort from the participant. In addition, clinical trials with different populations were included, such as traumatic brain injury [21, 23], arteriovenous malformation [23], and cerebral abscess [23]; and clinical outcomes were assessed by measurement instruments that measure different constructs (10 Meter Walk Test, Six Minute Walk Test, and Functional Ambulation Category included in the walking distance analysis). Different intervention characteristics (passive versus active and populations mixed) may have different effects on functional outcomes, so careful analysis of the impact of these differences is crucial. This evidence is important for healthcare professionals, policymakers, consumers, researchers, and others with an interest in this topic.

Therefore, the research questions for this systematic review were as follows:

1. Does cycling using FEST alone improve strength, balance, trunk control, walking speed, walking distance, and activities of daily living compared to no intervention or placebo in individuals after subacute stroke?
2. Does cycling using FEST combined with exercise programs improve strength, balance, trunk control, walking speed, walking distance, and activities of daily living compared to exercise programs in individuals after subacute stroke?
3. Does cycling using FEST alone improve strength, balance, trunk control, walking speed, walking distance, and activities of daily living compared to an exercise program in individuals after subacute stroke?

## Results

### Flow of studies through the review

**Fig. 1:**
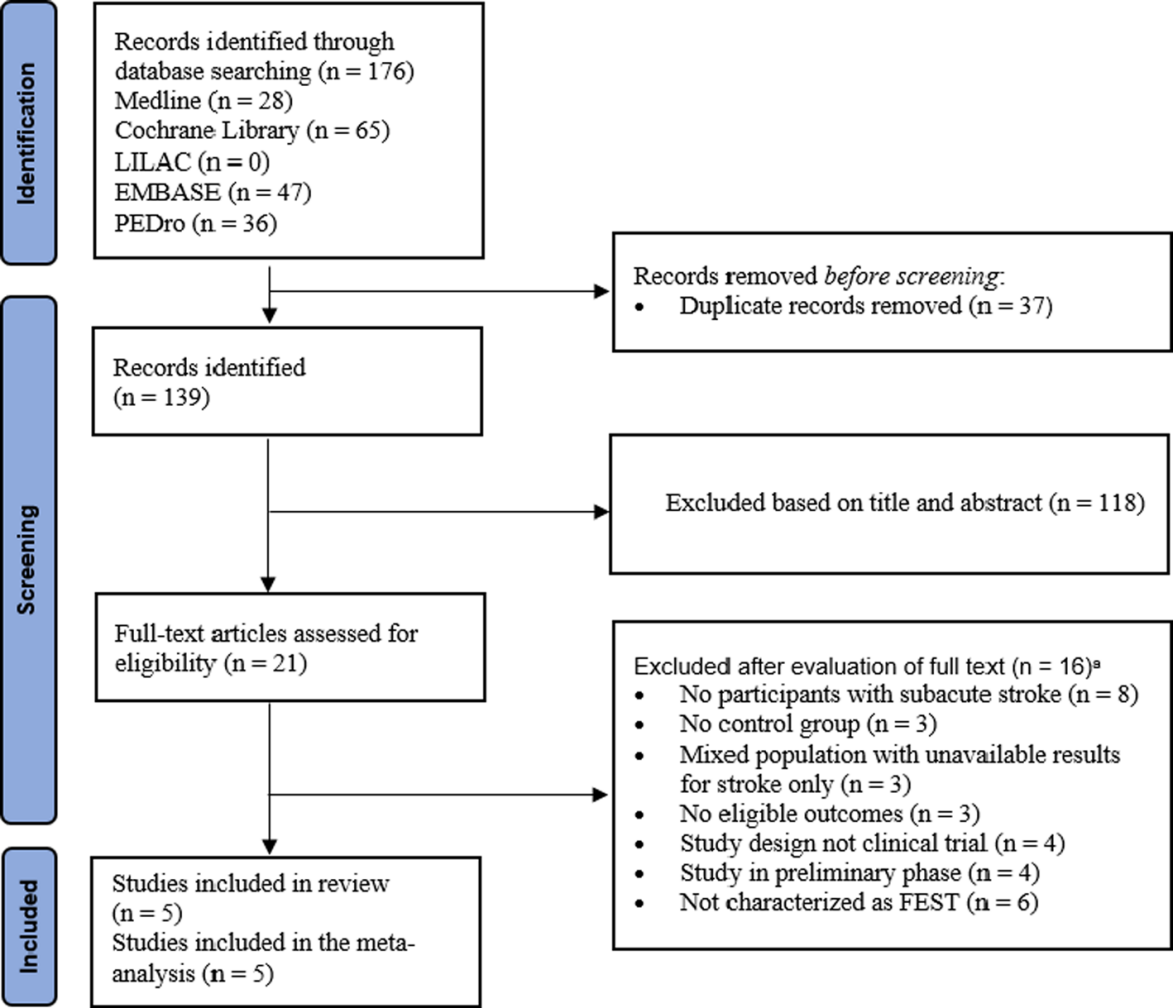
Flow of trials through the review. ^a^Trials may have been excluded for failing to meet more than one inclusion criterion

The search strategy identified 176 studies. After screening titles and abstracts, 21 potentially relevant studies were identified, and their full texts were retrieved. Sixteen studies failed to meet the inclusion criteria (see Additional file 1: Appendix S1 in the addenda for a summary of the excluded studies) leaving five studies included in the review. An outline of the screening and reviewing process is shown in Fig. 1.

### Characteristics of studies

The five studies involved 187 participants and investigated cycling using FEST combined with exercise programs or alone to improve strength (*n* = 2) [17, 18], balance (*n* = 1) [24], trunk control (*n* = 1) [18], walking speed (*n* = 3) [17–19], and walking distance (*n* = 3) [18, 19, 24], activities of daily living (*n* = 3) [19, 24, 25], after early subacute stroke. Additional information was requested from the authors of one study [24], but no information was received. See Table 1 for details of the included trials.

**Table 1:** Characteristics of included trials (*n* = 5)

Study	Design	Participants	Intervention^a^		Outcome measures^b^
			Frequency and duration	Characteristics	
Bauer et al. [17]	RCT	*n* = 37 Age (*y*) = 61 (12) Time since stroke (days) = 52 (44) Early subacute phase	Exp: cycling using FEST alone 20 min × 3/wk x 4wk Con: cycling without functional electrical stimulation 20 min × 3/wk x 4wk End treatment (4wk)^c^ and 6wk	Affected leg Muscles: knee flexors and extensors Frequency: 20–60 Hz Pulse width: 300–450 µs Current: visible muscle contraction below the pain threshold	Motricity Index (0–100) 10MWT (m/s)
Ambrosini et al. [18]	RCT	*n* = 52 Age (*y*) = 74 (12) Time since stroke (days) = 16 (10) Early subacute phase 3wk and end treatment (6 wk)^c^, and 30 wk	Exp: cycling using FEST + exercise programs (stretching, muscular conditioning, exercises for trunk control, standing, cycling and walking, and upper limb rehabilitation) (20 min + 70 min) × 5/wk x 6wk Con: exercise programs (stretching, muscular conditioning, exercises for trunk control, standing, cycling and walking, and upper limb rehabilitation) 70 min × 5/wk x 6wk	Affected and unaffected leg Muscles: knee flexors, ankle dorsiflexors and plantar flexors Frequency: 20 Hz Pulse width: 400 µs Current: Affected leg: visible contraction under pain tolerance threshold Unaffected leg: above the sensory threshold	Motricity Index (0–100) 10MWT (m/s) 6MWT (m) Berg Balance Scale (0–56) Trunk Control Test (0–21)
Peri et al. [19]	RCT	*n* = 16 Age (*y*) = 74 (10) Time since stroke (days) = 15 (4) Early subacute phase	Exp: cycling using FEST + exercise programs (strength and stretching exercises, gait training, stairs, hand rehabilitation) (25 min + 50 min) × 5/wk x 3wk Con: exercise programs (strength and stretching exercises, gait training, stairs, hand rehabilitation) 75 min × 5/wk x 3wk End treatment (3 wk)^c^	Affected and unaffected leg Muscles: knee flexors and extensors, ankle dorsiflexors and plantar flexors Frequency: NR Pulse width: NR Current: Affected leg: maximum intensity tolerated Unaffected leg: visible muscle contraction	6MWT (m/s) 6MWT (m) FIM (18–126)
Lee et al. [24]	RCT	*n* = 16 Age (*y*) = 63 (14) Time since stroke (days) = 60 (43) Early subacute phase	Exp: cycling using FEST alone 30 min × 5/wk x 4wk Con: cycling without functional electrical stimulation 30 min × 5/wk x 4wk End treatment (4wk)^c^	Affected leg Muscles: hip extensor, knee flexors and extensors, ankle plantar flexors Frequency: 60 Hz Pulse width: 300 µs Current: maximal comfortably tolerated	6MWT (m) Berg Balance Scale (0 to 56) Korean-Modified Barthel Index (0—100)
Zhang et al. [25]	RCT	*n* = 66 Age (*y*) = 56 (11) Time since stroke (days) = 43 (5) Early subacute phase	Exp: cycling using FEST + exercise programs (exercise therapy, physical therapy, and traditional treatment) (30 min + NR) × 6/wk x 8wk Con: cycling with functional electrical stimulation sham + exercise programs (exercise therapy, physical therapy, and traditional treatment) (30 min + NR) × 6/wk x 8wk 4wk and end treatment (8wk)^c^	Affected leg Muscles: knee flexors and extensors, ankle dorsiflexors and plantar flexors Frequency: 15 a 50 Hz Pulse width: 200 a 300 µs Current: visible muscle contraction	Modified Barthel index (0 to 100)

*Con* control group, *Exp* experimental group, *FEST* functional electrical stimulation therapy, *FIM* functional independence measure, *NR* not reported, *RCT* randomized controlled trial, *10MWT* 10 Meter Walk Test, *6MWT* Six Minute Walk Test

^a^Only the groups related to the current study objectives are shown

^b^Only the outcomes relevant to this review are listed

^c^Time points considered in this systematic review

#### Quality

The mean PEDro score of the studies was 5.8 (range 4–7). PEDro criteria and scores for the included trials are shown in Table 2. All the trials had randomly allocated participants, had similar groups at baseline, and reported between-group differences and measures of variability. Two studies had blinded assessors (40%), and four studies (80%) had a dropout rate of < 15%. One study reported whether intention-to-treat analysis was performed (20%). Two studies reported the use of concealed allocation (40%), and there was no blinding of participants or therapists across the studies.

**Table 2:** PEDro scores of included trials (*n* = 5)

Study	Eligibility and source^a^	Random allocation	Concealed allocation	Groups similar at baseline	Participant blinding	Therapist blinding	Assessor blinding	< 15% lost to follow-up	Intention-to- treat analysis	Between-group difference reported point	Point estimate and variability reported	Total (0–10)
Bauer et al. [17]	N	Y	N	Y	N	N	Y	Y	Y	Y	Y	7
Ambrosini et al. [18]	Y	Y	Y	Y	N	N	N	Y	N	Y	Y	6
Peri et al. [19]	N	Y	Y	Y	N	N	Y	Y	N	Y	Y	7
Lee et al. [24]	Y	Y	N	Y	N	N	N	N	N	Y	Y	4
Zhang et al. [25]	Y	Y	N	Y	N	N	N	Y	N	Y	Y	5

*N* no, *Y* yes

^a^Relates to external validity and therefore does not contribute to the total score

#### Participants

The mean age of the participants ranged from 56 to 74 years of age. All studies [17–19, 24, 25] included participants in the early subacute post-stroke stage, the time of stroke ranged from 16 to 60 days. Mean baseline strength ranged from 22.5 to 79.9 points on the Motricity Index and one study [25] did not report baseline strength of participants. Mean baseline walking speed ranged from 0.61 m/s to 0.7 m/s, and three studies [17, 18, 25] did not report participants' baseline walking speed.

#### Intervention

The experimental intervention in all trials was cycling using FEST combined with exercise programs or alone. Studies comparing cycling using FEST combined with exercise programs versus no intervention were not found. Three studies [18, 19, 25] investigated the effectiveness of cycling using FEST combined with exercise programs. Two studies [17, 24] investigated the effectiveness of cycling using FEST alone versus exercise program. The studies examined the experimental intervention for 20–30 min, 3–6 days per week, for 3–8 weeks. Three studies [17, 24, 25] used functional electrical stimulation on the affected leg and two studies [18, 19] used functional electrical stimulation on both legs. Electrodes were positioned on the hip extensor [19], knee flexor [17–19, 24, 25], knee extensor [17, 19, 24, 25], dorsiflexor [18, 19, 25], and plantar flexor muscle groups [18, 19, 24, 25]. The frequency of functional electrical stimulation ranged from 15 to 60 Hz and the pulse width ranged from 200 to 450 µs; one study [19] did not report the electrostimulation parameters. All control groups of the included studies were composed of exercise programs such as muscle conditioning training, strengthening, trunk control exercises, stretching, gait training, and cycling without functional electrical stimulation lasting between 30 and 75 min. One study [25] did not report the duration of the exercise programs. The control group received exercise programs [18, 19], exercise programs associated with cycling with functional electrical stimulation sham [25], or cycling without functional electrical stimulation [17, 24].

#### Outcome measures

Of the included trials, all provided data for motor function and activity analyses. Motor function analyses consisted of two studies [17, 18] that measured strength using the Motricity Index, one study [24] that measured balance using the Berg Balance Scale, and the other study [18] that measured trunk control by the Trunk Impairment Scale. Activity analyses consisted of four studies [17–19, 24] that measured walking speed, three studies [18, 19, 24] measured walking distance using the Six Minute Walk Test, and three studies [19, 24, 25] measured activities of daily living.

### Effectiveness of cycling using FEST combined with exercise programs compared to exercise programs on motor function outcomes

#### Strength

The effectiveness of cycling using FEST combined with exercise programs on strength compared to exercise programs was examined in one trial [18] involving 52 participants. Moderate-quality evidence suggested that the mean difference of strength in the Motricity Index (0–100) was seven points (95% CI −2.70 to 16.70), which indicates that cycling using FEST combined with exercise programs provided similar benefits compared to exercise programs on strength.

#### Trunk control

The effectiveness of cycling using FEST combined with exercise programs on trunk control compared to exercise programs was examined in one trial [18] involving 52 participants. Moderate-quality evidence suggested that the mean difference of trunk control in the Trunk Impairment Scale (0–26) was nine points (95% CI 0.36–17.64), which indicates that cycling using FEST combined with exercise programs provided relevant benefits [26] compared to exercise programs on trunk control.

### Effectiveness of cycling using FEST combined with exercise programs compared to exercise programs on activity outcomes

#### Walking speed

**Fig. 2:**
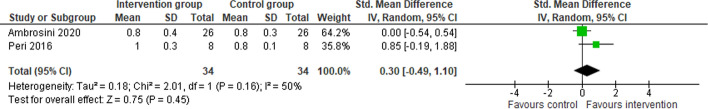
Standardized mean difference (95% CI) of the effectiveness of cycling using FEST combined with exercise programs compared to exercise programs for walking speed immediately after the intervention period

The effectiveness of cycling using FEST combined with exercise programs on walking speed compared to exercise programs was examined by pooling outcomes from two trials [18, 19] involving 68 participants. Low-quality evidence suggested that the standardized mean difference of walking speed was 0.3 in favor of cycling using FEST combined with exercise programs; however, the estimate was imprecise (95% CI −0.49 to 1.10, *I*^2^ = 50%), which indicates that cycling using FEST combined with exercise programs provided similar benefits compared to exercise programs on walking speed (Fig. 2).

#### Walking distance

**Fig. 3:**
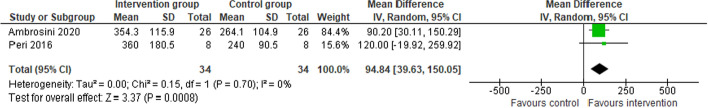
Mean difference (95% CI) of the effectiveness of cycling using FEST combined with exercise programs compared to exercise programs for walking distance immediately after the intervention period

The effectiveness of cycling using FEST combined with exercise programs on walking distance compared to exercise programs was examined by pooling outcomes from two trials [18, 19] involving 68 participants. Moderate-quality evidence suggested that the mean difference in walking distances in the Six Minute Walk Test was 94.84 m (95% CI 39.63–150.05, I = 0%), which indicates that cycling using FEST combined with exercise programs provided relevant benefits [27] compared to exercise programs on walking distance (Fig. 3).

#### Activities of daily living

**Fig. 4:**
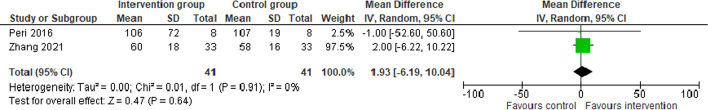
Mean difference (95% CI) of the effectiveness of cycling using FEST combined with exercise programs compared to exercise programs for activities of daily living immediately after the intervention period

The effectiveness of cycling using FEST combined with exercise programs on activities of daily living compared to exercise programs was examined by pooling outcomes from two trials [19, 25] involving 82 participants. Low-quality evidence suggested that the mean difference of activities of daily living in the Functional Independence Measure (18–126) was 1.93 points (95% CI −6.19 to 10.04, *I*^2^ = 0%), which indicates that cycling using FEST combined with exercise programs provided similar benefits compared to exercise programs on activities of daily living (Fig. 4).

### Effectiveness of cycling using FEST alone compared to exercise programs on motor function outcomes

#### Strength

The effectiveness of cycling using FEST alone compared to exercise programs on strength was examined by one trial [17] involving 37 participants. Moderate-quality evidence suggested that the mean difference of strength in the Motricity Index (0–100) was two points (95% CI −10.5 to 14.25), which indicates that cycling using FEST alone provided similar benefits compared to exercise programs on strength.

#### Balance

The effectiveness of cycling using FEST alone compared to exercise programs on balance was examined by one trial [24] involving 16 participants. Low-quality evidence suggested that the mean difference of balance in the Berg Balance Scale (0–56) was 4.5 points lower (95% CI −9.64 to 0.64), which indicates that cycling using FEST alone provided similar benefits compared to exercise programs on balance.

### Effectiveness of cycling using FEST alone compared to exercise programs on activity outcomes

#### Walking speed

**Fig. 5:**
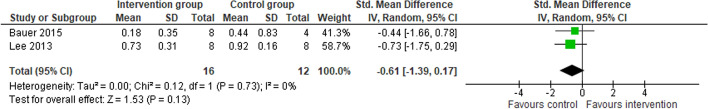
Standardized mean difference (95% CI) of the effectiveness of cycling using FEST alone compared to exercise programs for walking speed immediately after the intervention period

The effectiveness of cycling using FEST alone compared to exercise programs on walking speed was examined by pooling outcomes from two trials [17, 24] involving 28 participants. Low-quality evidence suggested that the standardized mean difference was -0.61 in favor of exercise programs (95% CI −1.39 to 0.17, *I*^2^ = 0%), which indicates that cycling using FEST alone provided similar benefits compared to exercise programs on walking speed (Fig. 5).

#### Walking distance

The effectiveness of cycling using FEST alone compared to exercise programs on walking distance was examined by on trial [24] involving 16 participants. Low-quality evidence suggested that the mean difference in walking distance in the Six Minute Walk Test was 65.25 m lower (95% CI −154.21 to 23.71), which indicates that cycling using FEST alone provided similar benefits compared to exercise programs on walking distance.

#### Activities of daily living

The effectiveness of cycling using FEST alone compared to exercise programs on activities of daily living was examined by one trial [24] involving 16 participants. Low-quality evidence suggested that the mean difference of activities of daily living in the Barthel index (0–100) was seven points lower (95% CI −7.23 to 3.23), which indicates that cycling using FEST alone provided similar benefits compared to exercise programs on activities of daily living.

### GRADE summaries

The overall quality of the evidence for each outcome in each comparison is shown in Additional file 1: Appendix S2.

### Publication Bias

Publication bias was not assessed by funnel plots due to the number of included studies < 10 [28].

## Discussion

The present study is the first systematic review with meta-analysis to investigate the effectiveness of cycling using FEST combined with exercise programs or alone on motor function and activity in post-stroke individuals in the subacute phase. The efficacy of cycling using FEST alone compared to no intervention or placebo could not be estimated due to insufficient studies. The effectiveness of cycling using FEST combined with exercise programs was superior or similar in motor function and activity outcomes in early subacute phase post-stroke individuals when compared to exercise programs alone. Moderate-quality evidence demonstrated effectiveness in favor of cycling using FEST combined with exercise programs for trunk control and walking distance compared to exercise programs in early subacute post-stroke. Low- to moderate-quality evidence demonstrated similar benefits on the other outcomes between cycling using FEST combined with exercise programs compared to exercise programs. Low- to moderate-quality evidence demonstrated that the effectiveness of cycling using FEST alone compared to exercise programs was similar in motor function and activity outcomes in early subacute post-stroke individuals.

Cycling using FEST combined with exercise programs compared to exercise programs demonstrated an effectiveness of nine points on the trunk impairment scale and 94.84 m in walking distance. These results are superior to the minimum detectable change for post-stroke individuals in the subacute phase of 3.5 points in the trunk impairment scale [26] and 60.98 m in the Six Minute Walk Test [27]. It is important to emphasize that the trunk control result was obtained through only one test and must be interpreted with caution. The benefits in the walking distance corroborate previous studies [20, 29] where the improvement was attributed to possible neural adaptation such as from functional electrical stimulation.

This review demonstrated similar benefits of cycling using FEST alone on strength and walking speed. These results could be explained by the reason that the participants had baseline strength measurements above the normality standard (Motricity Index > 54.3 points) [18, 19] and most of the participants were able to walk independently [17, 24]. Furthermore, we observed that the studies varied regarding the stimulation parameters, especially regarding the muscle groups stimulated, therapist command, and intervention time. We hypothesized that functional electrical stimulation delivered to the hip, knee, and ankle muscle groups was more likely to result in improved outcomes related to strength and walking. Although the studies considered the three requirements that characterize FEST (active cycling; assisted by functional electrical stimulation; and therapist-guided), the conduct of the intervention varied among the trials. For example, two studies [18, 24] required the participant to maintain a predetermined cadence and one study [19] included passive cycling in the experimental group between time intervals possibly reducing training intensity. Finally, the intervention time of the trials was below recommended, ranging from 3 to 8 weeks, and may have influenced the success of cycling using FEST alone. A systematic review [30] recommended that interventions for post-stroke individuals aiming to improve mobility should have a minimum duration of 12 weeks.

The present systematic review showed no statistical difference in the effectiveness of cycling using FEST alone in improving balance. Differently, a recent systematic review [29] demonstrated the effectiveness of cycling with functional electrical stimulation. Probably, the inclusion of studies with instruments that assess functional mobility (Functional Ambulation Category and Performance-Oriented Mobility Assessment) for the synthesis of balance effectiveness may have contributed to the result found by the study [29]. We emphasize the presence of high heterogeneity in the balance outcome, probably due to the low sample size and low methodological quality of one of the included studies [24]. Moreover, the balance outcome considered studies that included the experimental group cycling with functional electrical stimulation as the only intervention and cycling with functional electrical stimulation combined with exercise programs, which may have influenced the result of the analysis.

Of the trials included in this review, three studies had high methodological quality [17–19] (PEDro > 6). The most prevalent sources of bias among the studies were the non-blinding of participants and therapists since blinding is hardly possible due to the characteristics of the intervention. Another source of bias was the lack of reporting of intention-to-treat analysis. The small sample size (average of 19 participants per group) and the no sample size calculation were issues that reduced the quality of evidence of the GRADE system.

Our review investigated FEST for training targeted lower limb movements in post-stroke individuals. Our focus extended to exploring cycling as a modality within FEST, investigating its implications for transferring motor skills to fundamental tasks such as standing and walking. Furthermore, we highlight that studies employing FEST show promising results in the rehabilitation of upper limb function, particularly in activities like reaching and grasping [31]. In this way, our review contributes to and proposes a comprehensive exploration of various outcomes and potential applications of FEST in the field of stroke rehabilitation and other neurological health conditions. Our review has some limitations, although the studies contemplated the requirements that characterize FEST, there were differences in the parameters used among the studies. In this way, we observed that the equipment used in all the studies included did not allow synchronous functional electrical stimulation with cycling, so the participant should maintain a cadence predetermined by the therapists. Thus, the development of equipment that allows synchronizing functional electrical stimulation with cycling would possibly promote an appropriate cadence for each participant, promoting benefits in motor function and activity outcomes. Furthermore, because the publication of studies preceded the definition of FEST, we did not find the term FEST in the abstract titles, a potential limitation in the search strategy and access to potentially eligible studies. Finally, another limitation was the lack of standardization in the motor impairment definition of the participants included in the studies.

In conclusion, this systematic review provides clinical insights into the use of cycling using FEST for early subacute stroke individuals. There is low- to moderate-quality evidence that cycling using FEST combined with exercise programs is effective in providing benefits, similar or superior, in motor function and activity when compared to exercise programs. Mainly, clinicians should therefore be confident in prescribing cycling using FEST for individuals in the early subacute phase, when the objective of the intervention is to increase trunk control and walking distance. There is low- to moderate-quality evidence that cycling using FEST alone promotes similar benefits in motor function and activity outcomes when compared to exercise programs. In this way, cycling using FEST may be an effective strategy to consider in improving motor function and activity outcomes for post-stroke individuals in the early subacute phase. Future studies should investigate samples with more severe motor impairment and equipment that provides synchronous muscle stimulation during cycling phases.

## Methods

The review is reported according to the Preferred Reporting Items for Systematic Reviews and Meta-Analyses (PRISMA) statement guidelines [32].

### Identification and selection of trials

Searches were conducted on MEDLINE (1946 to July 2022), Cochrane (2005 to July 2022), EMBASE (1947 to July 2022), Latin American and Caribbean Literature on Health Sciences (LILACS) (to July 2022), and Physiotherapy Evidence Database (PEDro) (to July 2022), databases for relevant studies without date or language restrictions. Search terms included words related *to stroke, electric stimulation therapy, cycling,* and *clinical trials*. See Additional file 1: Appendix S3 on the addenda for the full details of the search strategy.

Titles and abstracts were screened independently by two authors (WG and LC) to identify relevant trials. The method section of the retrieved studies was extracted and reviewed independently by two reviewers (WG and LC) using predetermined criteria (Box 1). Both reviewers were blinded to the manuscript title, authors, journal, and results. Disagreements or ambiguities were resolved by discussion with a third reviewer (LL). The Rayyan tool was used for the selection and registration of the database search.

### Assessment of characteristics of trials

#### Quality

The methodological quality of the included trials was assessed by extracting the PEDro scores from the Physiotherapy Evidence Database website. The PEDro scale is an 11-item scale designed for rating the methodological quality (internal validity and statistical information) of randomized trials. Each item, except for Item 1, contributes one point to the total score (range 0–10 points). Where a trial was not included in the database, it was scored by a reviewer who had completed the PEDro scale training tutorial and checked by a second reviewer.

#### Participants

Trials examining participants over 18 years of age, early (7 days to 3 months), and/or late (3–6 months) post-stroke in the subacute phase were included [9, 33]. The number of participants, age, time since stroke, and the outcomes of interest were recorded to assess the similarity of the studies.

#### Intervention

The experimental intervention should meet the following criteria [15]: Participants should actively perform lower limb cycling. The cycling should be assisted by functional electrical stimulation of at least two muscle groups in the affected limb. The therapist could guide the movement and could adjust the stimulation during the period of the intervention. FEST in combination with other interventions was also included. The control group was divided into no intervention (e.g., placebo or passive interventions) and exercise programs (e.g., standard care, gait training, cycling without functional electrical stimulation). The frequency and duration of sessions were recorded to assess the similarity of the studies.

#### Outcome measures

The outcomes of interest were motor function and activity. Motor function outcomes were defined according to the body function component and included strength measured by maximum force production or by composite scales of multiple lower limb muscle groups (i.e., Motor Index); balance obtained by validated and standardized instruments (i.e., Berg Balance Scale); and trunk control obtained by instruments that assess trunk function, sitting balance, or both (i.e., Trunk Impairment Scale). Activity outcomes were defined according to the activity component and included walking speed typically obtained by timed walking test, reported by a ratio between distance in meters and time in seconds; walking distance obtained by the maximum distance walked at usual speed for a predetermined time, usually for six minutes (i.e., Six Minute Walk Test); and activities of daily living obtained by validated and standardized instruments (i.e., Barthel Index and Functional Independence Measure) [34]. The timing of the measurements and the procedure used to measure the outcomes were recorded to assess the appropriateness of combining studies in a meta-analysis.

#### Data extraction and analysis

Information about the method (i.e., design, participants, intervention, and measures) and results (i.e., number of participants, mean and standard deviation of motor function, and activity-related outcomes) was extracted independently by two reviewers (WG and LC) and checked by a third reviewer (LL). Where information was unavailable in the published trials, details were requested from the corresponding author, or data were estimated using methods recommended in the Cochrane Handbook for Systematic Reviews of Interventions [28].

Post-intervention changes were used to obtain the pooled estimate of the intervention effectiveness using a random-effects model. A visual inspection of the distribution of effect sizes was performed using the forest plot, and the *I*^2^ value was calculated to indicate the proportion of variance that was due to heterogeneity [35]. Values of *I*^2^ > 50% are indicative of high heterogeneity. The analyses were performed using Review Manager Version 5.3 (The Nordic Cochrane Centre, Copenhagen, Denmark). Post-intervention scores were used to calculate the mean difference (MD) when outcomes were measured in the same measurement units. When outcomes were measured on different scales they were used to calculate the standardized mean difference (SMD). Data for each outcome were reported as the pooled difference between the intervention and control groups and their 95% confidence interval (CI).

The Grading of Recommendations Assessment, Development, and Evaluation (GRADE) system was used to summarize the overall quality of evidence for each outcome. The GRADE system ranges from high to very low quality [36]. This review classified the evidence starting at the high-quality level and downgraded it one point whenever one of the following prespecified criteria was present: risk of bias (defined as > 50% of clinical trials with a PEDro score < 6); inconsistency (I^2^ > 50%); indirectness (> 50% of the participants were not related to the trial’s target audience); imprecision (< 400 participants in the comparison for continuous outcomes and > 300 participants for categorical outcomes); and publication bias (will be assessed using a funnel plot in the presence of > 10 studies in the same comparison). Two reviewers (WG and LC) evaluated the quality of evidence using the GRADE system, with possible disagreements resolved by discussion with a third reviewer (LL).

### Supplementary Information

**Additional file 1: Appendix S1.** Excluded papers. **Appendix S2.** GRADE summaries. **Appendix S3.** Search strategy.

## Data Availability

All data from this review are available in the PROSPERO database under the following accession number (CRD42022345282).
