# Supplementary material for: Cycling using functional electrical stimulation therapy to improve motor function and activity in post-stroke individuals in early subacute phase: a systematic review with meta-analysis
Source: Biomed Eng Online. 2024 Jan 2;23:1. doi: 10.1186/s12938-023-01195-8 (PMC10762955; doi:10.1186/s12938-023-01195-8)
Supplement: Supplementary file 1 — Additional file 1: Appendix S1. Excluded papers. Appendix S2. GRADE summaries. Appendix S3. Search strategy. [file 12938_2023_1195_MOESM1_ESM.docx]

**Appendix S1.** Excluded papers

|  | **Reasons for exclusion** | | | | | | |
| --- | --- | --- | --- | --- | --- | --- | --- |
| Studies | 1 | 2 | 3 | 4 | 5 | 6 | 7 |
| Ferrante et al (2008)^1^ |  |  |  |  |  |  | ✓ |
| Ambrosini et al (2020)^2^ |  | ✓ |  |  |  |  |  |
| Ofori et al (2019)^3^ |  |  |  | ✓ | ✓ |  |  |
| Jin et al (2013)^4^ | ✓ |  |  | ✓ | ✓ | ✓ |  |
| Jin et al (2012)^5^ | ✓ |  |  |  |  | ✓ |  |
| Janssen et al (2008)^6^ | ✓ |  |  |  |  |  |  |
| Au et al (2019)^7^ | ✓ | ✓ |  | ✓ | ✓ |  | ✓ |
| Ambrosini et al (2011)^8^ |  |  | ✓ |  |  |  |  |
| Pinheiro et al (2021)^9^ |  |  |  |  |  |  | ✓ |
| Katz-Leurer et al (2006)^10^ |  |  |  |  |  | ✓ | ✓ |
| Shariata et al (2021)^11^ | ✓ |  |  |  |  |  |  |
| Yang et al (2014)^12^ | ✓ |  |  |  |  |  | ✓ |
| Ambrosini et al (2012)^13^ |  |  | ✓ |  |  |  |  |
| Lo et al (2012)^14^ | ✓ |  |  |  |  |  |  |
| Alon et al (2011)^15^ | ✓ | ✓ |  |  | ✓ | ✓ | ✓ |
| de Sousa et al (2016)^16^ |  |  | ✓ |  |  |  |  |

1. No participants with subacute stroke (n = 8)
2. No control group (n = 3)
3. Mixed population with unavailable results for stroke only (n = 3)
4. No eligible outcomes (n = 3)
5. Study design not clinical trial (n = 4)
6. Study in preliminary phase (n = 4)
7. Not characterized as FEST (n = 6)

**References**

1. Ferrante S, Pedrocchi A, Ferrigno G, Molteni F. Cycling induced by functional electrical stimulation improves the muscular strength and the motor control of individuals with post-acute stroke. *Eur J Phys Rehabil Med*. 2008;44(2):159-167.

2. Ambrosini E, Parati M, Peri E, De Marchis C, Nava C, Pedrocchi A. Changes in leg cycling muscle synergies after training augmented by functional electrical stimulation in subacute stroke survivors: a pilot study. *J Neuroeng Rehabil*. 2020;17(1 CC-Stroke):35. doi:10.1186/s12984-020-00662-w

3. Ofori EK, Frimpong E, Ademiluyi A, Olawale OA. Ergometer cycling improves the ambulatory function and cardiovascular fitness of stroke patients-a randomized controlled trial. *J Phys Ther Sci*. 2019;31(3):211-216. doi:10.1589/JPTS.28.211

4. Jin H, Jiang Y, Wei Q, Chen L, Ma G. Effects of aerobic cycling training on cardiovascular fitness and heart rate recovery in patients with chronic stroke. *NeuroRehabilitation*. 2013;32(2):327-335. doi:10.3233/NRE-130852

5. Jin H, Jiang Y, Wei Q, Wang B, Ma G. Intensive aerobic cycling training with lower limb weights in Chinese patients with chronic stroke: discordance between improved cardiovascular fitness and walking ability. *Disabil Rehabil*. 2012;34(19):1665-1671. doi:10.3109/09638288.2012.658952

6. Janssen TW, Beltman JM, Elich P, et al. Effects of electric stimulation-assisted cycling training in people with chronic stroke. *Arch Phys Med Rehabil*. 2008;89(3):463-469. doi:10.1016/J.APMR.2007.09.028

7. Au CY, Mehra P, Leung KWC, Tong RKY. Effects of Electromyographically-driven Neuromuscular Stimulatio Cycling System on the Lower-Limb of Stroke Survivors. *IEEE Int Conf Rehabil Robot*. 2019;2019:300-304. doi:10.1109/ICORR.2019.8779541

8. Ambrosini E, Ferrante S, Pedrocchi A, Ferrigno G, Molteni F. Cycling induced by electrical stimulation improves motor recovery in postacute hemiparetic patients: a randomized controlled trial. *Stroke*. 2011;42(4):1068-1073. doi:10.1161/STROKEAHA.110.599068

9. Da Rosa Pinheiro DR, Cabeleira MEP, Da Campo LA, Correâ PS, Blauth AHEG, Cechetti F. Effects of aerobic cycling training on mobility and functionality of acute stroke subjects: A randomized clinical trial. *NeuroRehabilitation*. 2021;48(1):39-47. doi:10.3233/NRE-201585

10. Katz-Leurer M, Sender I, Keren O, Dvir Z. The influence of early cycling training on balance in stroke patients at the subacute stage. Results of a preliminary trial. *Clin Rehabil*. 2006;20(5):398-405. doi:10.1191/0269215505CR960OA

11. Shariat A, Nakhostin Ansari N, Honarpishe R, et al. Effect of cycling and functional electrical stimulation with linear and interval patterns of timing on gait parameters in patients after stroke: a randomized clinical trial. *Disabil Rehabil*. 2021;43(13):1890-1896. doi:10.1080/09638288.2019.1685600

12. Yang HC, Lee CL, Lin R, et al. Effect of biofeedback cycling training on functional recovery and walking ability of lower extremity in patients with stroke. *Kaohsiung J Med Sci*. 2014;30(1):35-42. doi:10.1016/J.KJMS.2013.07.006

13. Ambrosini E, Ferrante S, Ferrigno G, Molteni F, Pedrocchi A. Cycling induced by electrical stimulation improves muscle activation and symmetry during pedaling in hemiparetic patients. *IEEE Trans Neural Syst Rehabil Eng*. 2012;20(3):320-330. doi:10.1109/TNSRE.2012.2191574

14. Lo HC, Hsu YC, Hsueh YH, Yeh CY. Cycling exercise with functional electrical stimulation improves postural control in stroke patients. *Gait Posture*. 2012;35(3):506-510. doi:10.1016/J.GAITPOST.2011.11.017

15. Alon G, Conroy VM, Donner TW. Intensive training of subjects with chronic hemiparesis on a motorized cycle combined with functional electrical stimulation (FES): a feasibility and safety study. *Physiother Res Int*. 2011;16(2):81-91. doi:10.1002/PRI.475

16. de Sousa DG, Harvey LA, Dorsch S, Leung J, Harris W. Functional electrical stimulation cycling does not improve mobility in people with acquired brain injury and its effects on strength are unclear: a randomised trial. *J Physiother*. 2016;62(4):203-208. doi:10.1016/J.JPHYS.2016.08.0

**Appendix S2.** GRADE summaries

| **Cycling using FEST combined with exercise programs compared to exercise programs** | | | | | |
| --- | --- | --- | --- | --- | --- |
| **Patient or population: subacute post-stroke patients**  **Intervention: Cycling using FEST combined with exercise programs**  **Comparison: exercises program** | | | | | |
| **Outcomes** | **Illustrative comparative risks* 95% CI** | | **Number of participants (trials)** | **Quality of evidence (GRADE)** | **Comments** |
|  | **Assumed risk** | **Assumed risk** |  |  |  |
|  | **Exercises program** | **Cycling using FEST** |  |  |  |
| Strength  Motricity Index (0 to 100) | The mean strength after the intervention in the control groups was 81 points | The mean strength in the experimental group was seven points higher (-2.70 to 16.20) | 52 participants (1 trial) | ⊕⊕⊕⊝ **Moderate**^1^ |  |
| Trunk control  Trunk Impairment Scale (0 to 26) | The mean trunk control after the intervention in the control groups was 89 points | The mean trunk control in the experimental group was nine points higher (0.36 to 17.64) | 52 participants (1 trial) | ⊕⊕⊕⊝ **Moderate**^1^ |  |
| Walking speed  10MWT (m/s) and 6MWT(m/s) | The mean walking speed after the intervention in the control groups was 0.8 m/s | The mean walking speed in the experimental group was 0.3 higher (–0.49 to 1.10) | 68 participants (2 trial) | ⊕⊕⊝⊝ **Low**^1,2^ |  |
| Walking distance  6MWT (m) | The mean walking distance after the intervention in the control groups was 252 meters | The mean walking distance in the experimental group was 94.84 meters higher (39.63 to 150.05) | 68 participants (2 trial) | ⊕⊕⊕⊝ **Moderate**^1^ |  |
| Activities of daily living  Functional Independence Measure (18 to 126) | The mean activities of daily living after the intervention in the control groups was 83 points | The mean activities of daily living in the experimental group was 1.93 points higher (-6.19 to 10.04) | 82 participants (2 trial) | ⊕⊕⊝⊝ **low**^1,3^ |  |
| ^1^ Downgraded due to imprecision  ^2^ Downgraded due to inconsistency  ^3^ Downgraded due to risk of bias | | | | | |

| **Cycling using FEST alone compared with exercises program** | | | | | |
| --- | --- | --- | --- | --- | --- |
| **Patient or population: subacute post-stroke patients**  **Intervention: Cycling using FEST alone**  **Comparison: exercises program** | | | | | |
| **Outcomes** | **Illustrative comparative risks* 95% CI** | | **Number of participants (trials)** | **Quality of evidence (GRADE)** | **Comments** |
|  | **Assumed risk** | **Assumed risk** |  |  |  |
|  | **Exercises program** | **Cycling using FEST** |  |  |  |
| Strength  Motricity Index (0 to 100) | The mean strength after the intervention in the control groups was 34 points | The mean strength in the experimental group was two points higher (-10.5 to 14.25) | 37 participants (1 trial) | ⊕⊕⊕⊝ **Moderate**^1^ |  |
| Balance  Berg balance Scale (0 to 56) | The mean balance after the intervention in the control groups was 51 points | The mean overall improvement in the experimental group was 4.5 points lower (-9.64 to 0.64) | 16 participants (1 trial) | ⊕⊕⊝⊝ **low**^1,3^ |  |
| Waling speed  10MWT (m/s) and 6MWT(m/s) | The mean walking speed after the intervention in the control groups was 0.8 m/s | The mean walking speed in the experimental group was 0.61 m/s lower (–1.39 to 0.17) | 28 participants (2 trial) | ⊕⊕⊝⊝ **Low**^1,3^ |  |
| Walking distance  6MWT (m) | The mean walking distance after the intervention in the control groups was 331.5 meters | The mean overall improvement in the experimental group was 65.25 points lower (-154.21 to 23.71) | 16 participants (1 trial) | ⊕⊕⊝⊝ **low**^1,3^ |  |
| Activities of daily living  Barthel index (0 to 100) | The mean activities of daily living after the intervention in the control groups was 76 points | The mean overall improvement in the experimental group was seven points lower (-7.23 to 3.23) | 16 participants (1 trial) | ⊕⊕⊝⊝ **low**^1,3^ |  |
| ^1^ Downgraded due to imprecision  ^2^ Downgraded due to inconsistency  ^3^ Downgraded due to risk of bias | | | | | |

**Appendix S3.** Search strategy

**Medline (Ovid 1946)**

1. cerebrovascular disorders/ or exp basal ganglia cerebrovascular disease/ or exp brain ischemia/ or exp carotid artery diseases/ or expintracranial arterial diseases/ or exp intracranial arteriovenous malformations/ or exp "intracranial embolism and thrombosis"/ or expintracranial hemorrhages/ or stroke/ or exp brain infarction/ (stroke$ or cva or poststroke or post-stroke).tw.
2. brain injuries/ or brain injury, chronic/
3. (stroke$ or cva or poststroke or post-stroke or cerebrovasc$ or cerebral vascular).tw.
4. ((cerebral or cerebellar or brain$ or vertebrobasilar) adj5 (infarct$ or isch?emi$ or thrombo$ or emboli$ or apoplexy)).tw.
5. ((cerebral or brain or subarachnoid) adj5 (haemorrhage or hemorrhage or haematoma or hematoma or bleed$)).tw.
6. exp hemiplegia/ or exp paresis/
7. (hemipar$ or hemipleg$ or brain injur$).tw.
8. Gait Disorders, Neurologic/
9. 1 or 2 or 3 or 4 or 5 or 6 or 7 or 8
10. Functional electrical [stimulation.mp](http://stimulation.mp/).
11. Fes.mp.
12. Electric Stimulation Therapy/ or [electrostimulation.mp](http://electrostimulation.mp/).
13. [Electrotherapy.mp](http://electrotherapy.mp/).
14. Functional electrical stimulation [therapy.mp](http://therapy.mp/).
15. FEST.mp.
16. NMES.mp.
17. Neuromuscular electrical [stimulation.mp](http://stimulation.mp/).
18. 10 or 11 or 12 or 13 or 14 or 15 or 16 or 17
19. Cycling.mp. or Bicycling/
20. Bicycl* OR Cycling OR cycl*
21. Ergometry/ or [ergometer.mp](http://ergometer.mp/).
22. 19 or 20 or 21
23. Randomized Controlled Trials/ or random allocation/ or Controlled Clinical Trials/ or control groups/ or clinical trials/ or clinicaltrials, phase i/ or clinical trials, phase ii/ or clinical trials, phase iii/ or clinical trials, phase iv/
24. double-blind method/ or single-blind method/ or cross-overstudies/ or Program Evaluation/ or meta-analysis/
25. (randomized controlled trial or controlled clinical trial or clinical trial or meta analysis).pt.
26. random$.tw.
27. (controlled adj5 (trial$ or stud$)).tw.
28. (clinical$ adj5 trial$).tw.
29. ((control or treatment or experiment$ or intervention) adj5 (group$ or subject$ or patient$)).tw.
30. (quasi-random$ or quasi random$ or pseudo-random$ or pseudo random$).tw.
31. ((control or experiment$ or conservative) adj5 (treatment ortherapy or procedure or manage$)).tw.
32. 23 or 24 or 25 or 26 or 27 or 28 or 29 or 30 or 31
33. 9 and 18 and 22 and 32

**EMBASE**

**1º Health condition**

('cerebrovascular accident'/exp OR ‘acute cerebrovascular lesion’ OR ‘acute focal cerebral vasculopathy’ OR ‘acute stroke’ OR ‘apoplectic stroke’ OR ‘apoplexia’ OR ‘apoplexy’ OR ‘blood flow disturbance’ OR ‘brain accident’ OR ‘brain attack’ OR ‘brain blood flow disturbance’ OR ‘brain insult’ OR ‘brain insultus’ OR ‘brain vascular accident’ OR ‘cerebral apoplexia’ OR ‘cerebral insult’ OR ‘cerebral stroke’ OR ‘cerebral vascular accident’ OR ‘cerebral vascular insufficiency’ OR ‘cerebro vascular accident’ OR ‘cerebrovascular arrest’ OR ‘cerebrovascular failure’ OR ‘cerebrovascular injury’ OR ‘cerebrovascular insufficiency’ OR ‘cerebrovascular insult’ OR ‘cerebrum vascular accident’ OR ‘cryptogenic stroke’ OR ‘CVA’ OR ‘insultus cerebralis’ OR ‘ischaemic seizure’ OR ‘ischemic seizure’ OR ‘stroke’ OR ‘thrombotic stroke’)

'hemiplegia'/exp

'paresis'/exp

**2º Intervention**

('electrotherapy'/exp OR ‘electric stimulation therapy’ OR ‘electro therapy’ OR ‘electrostimulation therapy’ OR ‘therapeutic electric stimulation’ OR ‘therapeutic electrical stimulation’ OR ‘therapeutic electro-stimulation’ OR ‘therapeutic electrostimulation’)

('electrostimulation'/exp OR ‘electric field stimulation’ OR ‘electric stimulation’ OR ‘electrical stimulation’ OR ‘electro stimulation’ OR ‘electrostimulus’ OR ‘galvanostimulation’)

('functional electrical stimulation'/exp OR ‘FES’ OR ‘functional electric stimulation’ OR ‘functional electrostimulation’)

('neuromuscular electrical stimulation'/exp OR ‘neuromuscular electric stimulation’ OR ‘neuromuscular electricostimulation’ OR ‘NMES’)

**3º Intervention**

'cycling'/exp OR ‘bicycling’

'ergometer'/exp OR ‘ergometers’ OR ‘stress exercise trolley’

**4º Study Design**

'clinical trial'/exp OR 'randomized controlled trial'/exp OR 'controlled clinical trial (topic)'/exp

**PEDro**

Abstract & Title: Stroke AND Cycling

Therapy: [No appropriate value in this field]

Problem: [No appropriate value in this field]

Body Part: [No appropriate value in this field]

Subdiscipline: [No appropriate value in this field]

Topic: [No appropriate value in this field]

Method: Clinical trial

When Searching: Match all Search terms (AND)

**Lilacs**

(mh:("Acidente Vascular Cerebral")) OR (mh:(Stroke)) OR (mh:("Accidente Cerebrovascular")) OR (tw:(AVC)) OR (tw:(“AVC Agudo”)) OR (tw:(AVE)) OR (tw:(“Acidente Cerebral Vascular”)) OR (tw:(“Acidente Cerebrovascular”)) OR (tw:(“Acidente Vascular Cerebral” Agudo)) OR (tw:(“Acidente Vascular Encefálico”)) OR (tw:(“Acidente Vascular do Cérebro”)) OR (tw:(“Acidentes Cerebrais Vasculares”)) OR (tw:(“Acidentes Cerebrovasculares”)) OR (tw:(“Apoplexia Cerebral”)) OR (tw:(“Acidentes Vasculares Cerebrais”)) OR (tw:(Apoplexia)) OR (tw:(“Icto Cerebral”)) OR (tw:(“Ictus Cerebral”)) OR (tw:(exC10.228.140.300.775$)) OR (tw:(exC14.907.253.855$))

(mh:(“Estimulação Elétrica”)) OR (mh:(“Electric Stimulation”)) OR (mh:(“Estimulación Eléctrica”)) OR (tw:(“Functional Electrical Stimulation”)) OR (tw:(FES)) OR (tw:(NMES)) OR (tw:( “neuromuscular electrical stimulation”)) OR (tw:( exE05.723.402$))

(mh:(Ciclismo)) OR (mh:(Bicycling)) OR (mh:(Ciclismo))

**CENTRAL**

#1 [mh ^"cerebrovascular disorders"] or [mh "basal ganglia cerebrovascular disease"] or [mh "brain ischemia"] or [mh "carotid artery diseases"] or [mh "intracranial arterial diseases"] or [mh “intracranial arteriovenous malformations”] or [mh "intracranial embolism and thrombosis"] or [mh "intracranial hemorrhages"] or [mh ^stroke] or [mh "brain infarction"]

#2 [mh ^"brain injuries"] or [mh ^"brain injury, chronic"]

#3 (stroke or cva or poststroke or "post-stroke" or cerebrovasc* or cerebral next vasc*):ti,ab,kw

#4 ((cerebral* or cerebell* or brain* or vertebrobasilar) near/5 (isch*emi* or infarct* or thrombo* or emboli* or apoplexy*)):ti,ab,kw

#5 ((brain* or cerebral* or subarachnoid) near/5 (haemorrhage* or hemorrhage* or haematoma* or hematoma* or bleed*)):ti,ab,kw

#6 [mh ^hemiplegia] or [mh paresis]

#7 (hemipleg* or hemipar* or paresis or paretic or brain next injur*):ti,ab,kw

#8 [mh ^"gait disorders, neurologic"]

#9 #1 or #2 or #3 or #4 or #5 or #6 or #7 or #8

#10 MeSH descriptor: [Electric Stimulation Therapy] explode all trees

#11 (FES or "Functional electrical stimulation" or "Functional electrical stimulation therapy" or FEST or NMES or "Neuromuscular electrical stimulation"):ti,ab,kw

#12 #10 or #11

#13 (Cycling or Bicycling or Cycle or Bicycle or Pedaling or Ergometer or Ergometry or "Cycle training"):ti,ab,kw

#14 #9 and #12 and #13
